# Lessons learned from multisite implementation and evaluation of Project SHARE, a teen health information literacy, empowerment, and leadership program

**DOI:** 10.5195/jmla.2019.351

**Published:** 2019-01-01

**Authors:** Alla Keselman, Rachel Anne Chase, Jennifer Rewolinski, Yulia Chentsova Dutton, Janice E. Kelly

**Affiliations:** Senior Social Science Analyst, Division of Specialized Information Services, National Library of Medicine, Bethesda, MD, keselmana@nih.gov; Health Educator Consultant, Office of Health Equity, Florida Department of Health in Hillsborough, Gainesville, FL, rachel.anne.chase@gmail.com; MS Student, University of Maryland School of Nursing, Baltimore, MD, jennifer.rewolinski@gmail.com; Associate Professor, Department of Psychology, Georgetown University, Washington, DC, yuliacd@gmail.com; Acting Deputy Associate Director, Division of Specialized Information Services, and Chief, Outreach and Special Populations Branch, National Library of Medicine, Bethesda, MD, janice.kelly@nih.gov

## Abstract

**Background:**

This case study describes the implementation and evaluation of a multisite teen health information outreach program. The objectives of the program were to increase health knowledge, health information literacy, interest in health careers, community engagement, and leadership skills of teens in disadvantaged communities.

**Case Presentation:**

Teens at six sites across the country participated in a multi-week curriculum that focused on various aspects of health literacy, information literacy, and leadership. Lesson topics addressed personal health, social determinants of health, information quality, and communication and advocacy skills. Program evaluation included both quantitative and qualitative components and focused on multiple knowledge and skills outcome variables. Results suggested that while teens at all sites showed improvement, particularly with respect to engagement and interest in the topics, the degree of gains in knowledge and information literacy measures varied significantly from site to site.

**Conclusion:**

On-site implementation planning, cohesive integration of added activities, and emphasis on retention can contribute to implementation and evaluation effectiveness. This work also underscores the limitation of a purely quantitative approach to capturing the impact of health information and stresses the importance of supplementing numerical scores and statistics with qualitative data.

## BACKGROUND

Helping adolescents navigate health information has many potential benefits. Many US adolescents, particularly those from disadvantaged backgrounds, have limited health literacy [1], which is associated with negative health behaviors and outcomes. As many lifetime health habits form during adolescence [2], it is important to target this age group in outreach efforts. Adolescence is also the time of career choices, and health information programs are a way to raise awareness about health careers with a goal to diversify the health care workforce. In addition, adolescents’ orientation toward the future and interest in social action make them potentially great partners for health-related community outreach and advocacy.

Supporting health information outreach programs for adolescents is an important mission of the National Library of Medicine (NLM). Programs typically aim to produce several outcomes. For example, two programs conducted in low-income, primarily minority schools—Vital Information for a Virtual Age (¡VIVA!) Peer Tutor Project (with the University of Texas Health Sciences Center at San Antonio) and the South Carolina Teen Health Leadership Program (with the Medical University of South Carolina)—trained groups of high school students to develop and conduct health information outreach in their schools and larger communities [3–5]. These programs had positive impacts on the participants’ health literacy, sense of empowerment, communication, leadership skills, and interest in health careers. ¡VIVA! also had an impact on the broader school community.

Health information outreach projects usually occur in community settings and are carried out by organizations with limited budgets, time, and staff, often without evaluation expertise. In a review of published studies evaluating health information outreach, the authors found that few evaluations involve pretest-posttest designs, test for statistical significance, or measure long-term project impacts [6]. Projects often report effectiveness in terms of numbers of attendees and rarely measure health knowledge, information literacy, and behaviors. Therefore, we recommended that outreach programs strengthen their evaluation planning and the breadth of their outcome measures (e.g., to include a range of information behaviors and attitudes).

## STUDY PURPOSE

This paper describes and evaluates the implementation of a health information outreach program aiming to improve health knowledge, information literacy, interest in health careers, community engagement, and leadership and communication skills of teens in disadvantaged communities. The program was a collaboration between NLM and the National Area Health Education Centers Organization (NAO). The protocol was approved by the National Institutes of Health Office of Human Subjects Research (survey) and the National Institute of Child Health and Human Development Institutional Review Board (focus groups).

## CASE PRESENTATION

### Project SHARE teen health literacy and leadership curriculum

The NLM-NAO collaboration involved an adaptation of the Student Health Advocates Redefining Empowerment (Project SHARE) health literacy and leadership curriculum that was developed by the Health Sciences and Human Services Library of the University of Maryland, Baltimore, with NLM funding. The six-module (nineteen individual lessons) program was a combination of lectures, group discussions, and student-led health promotion and advocacy activities (supplemental Appendix A) that aligns with the “National Health Education Standards.”

Module I introduced teens to health disparities and social determinants of health and prompted them to discuss concerns in their communities. Module II focused on health literacy, information seeking, and evaluation and built foundational skills for later advocacy projects. In module III, students learned about important strategies for maintaining personal health, such as prevention, awareness of family history, and doctor-patient communication. Module IV was about healthy nutrition, including food labels and meal planning. Modules V and VI focused on essential community health advocacy competencies: effective communication, leadership, health policy, and advocacy/outreach.

### National Library of Medicine (NLM)–National Area Health Education Centers Organization (NAO) Project SHARE adaptation and implementation process

To carry out the project, NLM contracted NAO to select six local Area Health Education Centers (AHECs) from low-income and minority communities that were interested in adapting and testing the curriculum:

- Boston: urban community
- Brooklyn-Queens-Long Island (BQLI): urban community
- Northeastern Colorado (NE CO): rural community
- Southwestern Colorado (SW CO): rural, Native American tribal territory
- Eastern Connecticut (E CT): Native American tribal territory
- Montana (MT) AHECs: rural, Native American tribal territory

While Project SHARE modules that were chosen for implementation varied somewhat among sites (supplemental Appendix A), all sites included an overview of health disparities and quality health information (modules I and II). All but one site emphasized introducing student participants to health or health information careers. Finally, all sites included additional student leadership activities that aimed to give opportunities to draw upon the information from earlier lessons. For example, students in Boston produced videos about the importance of cultural competence in health care, and students in Colorado participated in healthy cooking competitions that parents and community members attended. Sites also conducted field trips and hosted guest speakers. Staff from all sites participated in biweekly conference calls with one another and with NAO and NLM in which they shared experiences and ideas and discussed any needed adjustments.

### Participants

Table 1 shows the number of students participating in various evaluation activities at each site. The greatest challenge of the evaluation, common in community research settings, was obtaining data from all the students. In SW CO, a very-near car accident caused the pretest to be rescheduled. In NE CO, the program lead changed jobs soon after the posttest, and some of the posttests could not be located. In MT, the program was conducted in a school, and the AHEC staff inadvertently scheduled the posttest after the participating seniors graduated and could not be reached. Only paired data for the students who completed both pretests and posttests were used in the analysis. Because of low participant numbers in NE and SW CO, findings from those sites should be interpreted with caution.

**Table 1:** Participants in evaluation activities (numbers represent individual students)

Site	Started program	Completed program	Pretest	Posttest	Quantitative analysis (students with both pretest and posttest)	Focus group
Boston	14	8	14	9	8	8
BQLI	12	8	12	8	8	8
MT	16	16	12	11	8	13
NE CO	15	9	12	3	3	3
SW CO	12	11	10	7	6	3
E CT	26	26	21	24	20	18*
Total	95	78	81	62	53	53

BQLI=Brooklyn-Queens-Long Island; NE CO=Northeastern Colorado; SW CO=Southwestern Colorado; E CT=Eastern Connecticut; MT=Montana.

* Two groups.

### Evaluation instruments and procedure

Evaluation procedures and instruments were developed by an NLM evaluator in collaboration with the AHEC teams and included quantitative (survey) and qualitative (focus group) components in accordance with health information outreach evaluation guidelines [7, 8]. The quantitative component involved pretest and posttest surveys of variables aligned with our framework for evaluating health information outreach [5, 6]. The variables, organized into eight sections or clusters, were as follows (second-level bullets denote variables within the cluster):

- Cluster 1: Knowledge of health disparities and social determinants of health
  ○ Number of factors recognized as social determinants of health
  ○ Average proportion of possible explanations per recognized health determinant
  ○ Proportion of possible reasons explaining a local disparity
- Cluster 2: Understanding of the importance of knowing one’s family history
  ○ Understanding of health relevance of one’s family history
- Cluster 3: Knowledge of health risk factors
  ○ Knowledge of health risk factors one can control
  ○ Knowledge health risk factors one cannot control
- Cluster 4: Knowledge of preventive health
  ○ Awareness of diseases that are public health concerns in the United States
  ○ Average number of known preventive health measures per disease
  ○ Preventive measure recognition
- Cluster 5: Knowledge of nutrition
  ○ Knowledge of nutritional groups and the basics of food labels
- Cluster 6: Information evaluation skills
  ○ Recognition of information quality markers of a hoax website
  ○ Recognition of information quality markers of an authoritative website
  ○ Knowledge of general online information quality criteria
- Cluster 7: Awareness of quality health information resources
  ○ Awareness of quality health information sites
  ○ Awareness of MedlinePlus
- Cluster 8: Knowledge of and interest in health careers
  ○ Number of health occupations known
  ○ Average knowledge score per known health occupation
  ○ Number of health occupations of interest

Students at each site answered survey sections pertaining to lessons presented at their sites. The qualitative component involved post-project focus group discussion of students’ experience. The full survey can be found in supplemental Appendix B, and supplemental Appendix C contains detailed description of the outcome variables and their correspondence to Project SHARE lessons and survey questions.

### Survey coding and statistical analysis

Some of the variables were simple counts of correct responses to multiple choice questions. Others, such as explanations of social determinants of health or information quality criteria, involved narrative answers and were coded and scored by comparing students’ answers against gold standard models obtained from expert response or existing guidelines (e.g., Evaluating Internet Sources: A Library Resource Guide). Two coders coded a subset of all narrative data establishing “substantial” to “almost perfect” levels of inter-rater reliability as evidenced by Cohen’s kappa values >0.61 [9].

To reduce the number of comparisons while assessing the significance of variables across sites, continuous outcome variables from each cluster were included in repeated measure multivariate analysis of variance (MANOVA) accounting for participants being nested within sites. If MANOVA showed a significant main effect of time (pretest to posttest) or time by site interaction for a cluster, further analysis was performed to identify which variables drove the difference. In addition, we conducted paired sample *t*-tests for within-site comparisons. As the results specified which variables drove significant main or interaction effects, Bonferroni correction for multiple comparisons was not applied. Categorical variables were analyzed using McNemar tests. Details of the statistical analysis are described in supplemental Appendix D. Due to data attrition, the analyses are underpowered and, likely, overly conservative.

## QUANTITATIVE RESULTS: SURVEY ANALYSIS

There were some significant improvements in six out of eight clusters. However, they were limited to specific cluster variables and individual sites. A summary of statistically significant pretest to posttest improvements for clusters, overall variables across sites, and variables within individual sites is presented in Table 2. The findings suggest overall across-sites improvements in Cluster 4: Knowledge of preventive health and Cluster 6: Information evaluation skills. In addition, the analysis suggests significant time by site interactions, which indicates differential program impact on different sites, for all clusters except Cluster 3: Knowledge of health risk factors and Cluster 7: Awareness of quality health information resources.

**Table 2:** Summary of pretest-posttest improvements for individual sites

	Time	Time × Site	Boston	BQLI	MT	NE CO	SW CO	E CT
Knowledge of health disparities and social determinants of health		**S**						
Number of factors recognized as social determinants of health				S				S
Average proportion of possible explanations per recognized health determinant		S					S	
Proportion of possible reasons explaining a local disparity							S	S
Awareness of the importance of knowing one’s family health history		**S**						
Awareness of health relevance of one’s family history		S	NA	NA		NA	NA	S
Knowledge of health risk factors								
Knowledge of health risk factors one can control			NA	NA		NA	NA	S
Knowledge of health risk factors one cannot control			NA	NA		NA	NA	S
Knowledge of preventive health	**S**	**S**						
Awareness of diseases that are public health concern in the United States	S	S	NA	NA	S	NA	NA	
Average number of preventive health measures per disease			NA	NA		NA	NA	
Preventive measures recognition*	S		NA	NA		NA	NA	S
Knowledge of nutrition		**S**						
Knowledge of nutritional groups and the basics of food labels		S	NA	NA		NA	NA	S
Information evaluation skills	**S**	**S**						
Recognition of information quality markers of a hoax site			S					
Recognition of information quality markers of an authoritative site	S	S	S				S	
Knowledge of general online information quality criteria	S		S	S	S		S	S
Awareness of quality health information resources								
Awareness of quality health information sites								S
Awareness of MedlinePlus*	S			S				S
Knowledge and interest in health careers		**S**						
Number of health occupations known			NA		NA		S	S
Average knowledge score per known health occupation			NA		NA			S
Number of health occupations of interest		S	NA	S	NA			

Key: Time=Time (pre-post) effect, Time × Site=time by site interaction effect, S=Statistically significant at *p*<0.05, NA=not applicable, because the material was not covered.

Shaded rows indicate variables with overall significant Time or Time × Site interaction effects.

* Non-parametric variable analyzed via McNemar test separately from the rest of the cluster.

The greatest number of statistically significant improvements on individual variables occurred in the E CT site. For Boston, all the quantitative gains were for the three variables of the information evaluation skills cluster, reflecting the primary focus of that group’s curriculum. For BQLI and MT, quantitative gains were sparse. Finally, for the CO groups, the proportions of students who completed both pretests and posttests were so small that results’ interpretation is not warranted. Supplemental Appendix D provides additional details

## QUALITATIVE RESULTS: FOCUS GROUPS ANALYSIS

### Favorite aspects of the program

At all sites, favorite aspects of the program included hands-on activities and field trips. Students at E CO also mentioned empowering factors related to the focus on Native American health (e.g., “that [the program] made the Pequot health topics just as important as white people topics”).

### Understanding of social factors influencing community health

The degree to which students were able to talk about social and cultural influences on the health of their community differed greatly from site to site. For example, students in the MT and CO groups only talked about personal health-related factors (e.g., if people decide to exercise more, the community will be healthier). By contrast, students at the other three sites talked about local funding, policy, and historical trauma influences on health. For example, a student at E CT said, “[Before], ‘health disparities’ was just a white people word that the doctors use to blame us for always having diabetes and cancer because we are Indian. But now I understand what the words mean and why they are using it.”

### Leadership and perceived ability to influence social change

At most sites, the students felt that when it came to improving health, they could do things for themselves, but not for others. A student in MT said, “[I am] not comfortable enough to help people to change their lifestyle.” By contrast, students at BQLI made many positive comments about how the program influenced their perceived ability to undertake social action toward improving their community’s substance abuse and nutrition-related problems. Several students reported wanting to “go into leadership and policy” or “health management” because of the program. Students at E CT similarly discussed steps that they could undertake to improve community health via social change. For example, they described group discussions about building an additional rehabilitation facility on the reservation and collaborating with youth from surrounding tribes on making Tribal Council take them seriously.

### Oral communication and presentation skills

Students at BQLI, Boston, and E CT talked about the positive impact of the program on their oral communication skills. This included gaining positive experiences of talking to adults and being taken seriously and learning to adjust presentations to different audiences and occasions.

### Knowledge and interest in health careers

Students at BQLI, NE CO, Boston, and E CT commented on how participating in the program broadened their awareness of health-related occupations “beyond nurse/physician.” Several students in Boston, where most participants were bilingual, talked about medical interpreting. A student in E CT was excited to learn that “it is possible to be in the health field without dealing with blood.” E CT students also learned about careers in Native American health (e.g., positions at Indian Health Services). This produced a sense of empowerment and enthusiasm, as illustrated by this comment: “Usually G. and B. are super quiet, but as soon as we started talking about getting jobs to help the elders or drug addicts, they were louder than anyone else!” Many students at these four sites stated that participation in the program either reinforced or sparked their interest in health-related careers.

## DISCUSSION

While the general profile of the impact of health information outreach described in this paper is consistent with those of other NLM programs with adolescents [3–5], the evaluation, which includes a broad range of quantitative measures, is more comprehensive. Improving teens’ knowledge of health and health careers, information skills, and leadership skills via health information outreach programs is possible yet challenging. The same can be said about evaluating such programs in a comprehensive manner.

### Making a measurable difference with health information

Quantitative gains differed significantly across the projects. While all groups showed improvements on some quantitative outcome variables, sites showing no significant improvements across many or most of those variables were far too common. The qualitative analysis created a more nuanced picture, highlighting program benefits that were difficult to capture with numeric scores. These included the program’s positive impact on the participants’ leadership and communication skills, and participants’ beliefs in their abilities to improve their communities, particularly in BQLI and E CT.

### Evaluation considerations

The approach and instruments used in this evaluation are further-reaching and more sensitive to nuances of the program’s effects than is common in health information outreach. As such, they can provide knowledge about the impact of health information on various aspects of knowledge, skills, and attitudes [6]. Yet, our study suffers from limitations that are common in small-scale program evaluation in community settings. The biggest challenges were controlling implementation fidelity and obtaining data from all participants. Many challenges and limitations of this evaluation can be addressed in future implementations. However, methodological imperfections are an inherent characteristic of such projects. Knowing this should not preclude programs from striving to conduct evaluation and not preclude the field from creating resources for building an evidence base for effective health information outreach.

### Lessons learned

While it is difficult to make a conclusive statement as to why the six projects fared differently, one likely factor is the amount of time for planning and building program capacity. While the other programs started in February, the E CT program began the following May, benefitting from both the extra time and the experiences of the others. Strong preestablished relationships with the community also seemed to matter, easing the logistics of recruitment and implementation. Yet another important consideration was the strength of the connection between the lessons and the leadership activities, which provided motivation and cohesion.

Tension between adaptability and fidelity of implementation is unavoidable in health information outreach programs that use existing curricula. This study illustrates the challenges that such conflicting requirements pose for implementation and evaluation. However, we believe that more in-depth staff training, longer planning periods, student incentives to prevent attrition, and stronger association between information and action will strengthen such programs’ impact.

## SUPPLEMENTAL FILES

Appendix A Original Project SHARE and National Library of Medicine (NLM)-National Area Health Education Centers Organization (NAO) program sites’ implementation of its activities

Appendix B National Library of Medicine (NLM)-National Area Health Education Centers Organization (NAO) Project SHARE Evaluation Survey

Appendix C Survey sections and outcome variables

Appendix D Details of statistical analysis and results
