# Supplementary material for: Lessons learned from multisite implementation and evaluation of Project SHARE, a teen health information literacy, empowerment, and leadership program
Source: J Med Libr Assoc. 2019 Jan 1;107(1):72–9. doi: 10.5195/jmla.2019.351 (PMC6300228; doi:10.5195/jmla.2019.351)
Supplement: Appendix A [file jmla-107-72-s001.pdf]

JMLA Journal of the Medical Library Association

SC

Supplemental content to
J Med Libr Assoc. Jan;107(1):dx.doi.org/10.5195/jmla.2019.351
www.jmla.mlanet.org
© Keselman, Chase, Rewolinski, Chentsova Dutton, Kelly 2019

Lessons learned from multisite implementation and evaluation of Project SHARE, a teen health information literacy, empowerment, and leadership program

Alla Keselman, PhD, MA; Rachel Anne Chase, MPH; Jennifer Rewolinski, BS; Yulia Chentsova Dutton, PhD; Janice E. Kelly, MLS, FMLA

APPENDIX A

Original Project SHARE and National Library of Medicine (NLM)–National Area Health Education Centers Organization (NAO) program sites' implementation of its activities

	Boston	BQLI	NE CO	SW CO	E CT	MT
Module I: Overview of Health Disparities						
1.1 Introduction to health disparities	I	I	I	I	I	I
1.2 Social determinants of health	I	I	I	I	I	I
Module II: Quality Health Information						
2.1 Health literacy	I	I	I	I	I	I
2.2 Locating and evaluating health information	I	I	I	I	I	I
2.3 Cultural competence	I	I			I	I
Module III: Taking Charge of Your Health						
3.1 Family health history					I	I
3.2 Preventive health		I	I	I	I	I
3.3 Providers and doctors' appointments						I
Module IV: Smart Food Choices						
4.1 The importance of food labels				I	I	
4.2 Planning a nutritious meal			I	I	I	I
Module V: Crafting and Delivering the Message						
5.1 Designing effective presentations		I			I	I
5.2 Speaking with impact						P
5.3 The elevator speech						P
5.4 In your own words						I

JMLA Journal of the Medical Library Association

SC

Supplemental content to
J Med Libr Assoc. Jan;107(1):dx.doi.org/10.5195/jmla.2019.351
www.jmla.mlanet.org
© Keselman, Chase, Rewolinski, Chentsova Dutton, Kelly 2019

	**Boston**	**BQLI**	**NE CO**	**SW CO**	**E CT**	**MT**
Module VI: Promoting Health and Wellness in Your Community						
6.1 Introduction to policy		I				I
6.2 Introduction to advocacy		I				I
6.3 Leadership		I			I	
6.4 Designing effective poster					I	
6.5 Getting started with outreach					P	
Additional activities (not in original Project SHARE)						
Health information careers*	I					
Health careers		I	I	I		I
Health equity	I					

BQLI=Brooklyn-Queens-Long Island; NE CO=Northeastern Colorado; SW CO=Southwestern Colorado; E CT=Eastern Connecticut; MT=Montana
I=implemented; P=partially implemented.
* Health sciences librarianship, health communication.
