# Supplementary material for: Lessons learned from multisite implementation and evaluation of Project SHARE, a teen health information literacy, empowerment, and leadership program
Source: J Med Libr Assoc. 2019 Jan 1;107(1):72–9. doi: 10.5195/jmla.2019.351 (PMC6300228; doi:10.5195/jmla.2019.351)
Supplement: Appendix B [file jmla-107-72-s002.pdf]

JMLA Journal of the Medical Library Association

SC

Supplemental content to
J Med Libr Assoc. Jan;107(1):dx.doi.org/10.5195/jmla.2019.351
www.jmla.mlanet.org
© Keselman, Chase, Rewolinski, Chentsova Dutton, Kelly 2019

Lessons learned from multisite implementation and evaluation of Project SHARE, a teen health information literacy, empowerment, and leadership program

Alla Keselman, PhD, MA; Rachel Anne Chase, MPH; Jennifer Rewolinski, BS; Yulia Chentsova Dutton, PhD; Janice E. Kelly, MLS, FMLA

APPENDIX B

National Library of Medicine (NLM)–National Area Health Education Centers Organization (NAO) Project SHARE evaluation survey*

Knowledge of health disparities and social determinants of health

1. In your opinion, which of the following makes a person more likely to be healthy?† (Check all that apply):

– Going to a doctor for regular checkups
– Being part of a family that owns a home‡
– Being part of a family that owns a car or other motorized transportation‡
– Living in a neighborhood that has well-lit sidewalks‡
– For kids, having adult supervision after school‡
– Not having asbestos or lead in the home
– Having most meals cooked at home
– Having more education
– Being generally happy
– Having trusted friends to talk to when upset

2A§. In Boston, if you live in Dorchester, Roxbury, or Mattapan, you are less likely to have good dental health. What factors, in your opinion, contribute to this?

2B§. Non-Hispanic black adults are at least 50% more likely to die of heart disease or stroke prematurely. What factors, in your opinion, contribute to this?

2C§. People of Hispanic descent, Native Americans, and Alaska Natives develop diabetes-related complications at almost double the rate of non-Hispanic whites. What factors, in your opinion, contribute to this?

Understanding of the importance of knowing one's family history in health

1. During a check-up visit, a doctor asks Mary about her parents' health. Most likely, the doctor:

– thinks she may know Mary's parents
– is being polite in order to make Mary feel at easy
– is looking for new patients
– **wants to know about things that may affect Mary's health (correct)**

---

* The survey was presented via SurveyMonkey tool, formatted for easy reading.
† Each answer option is followed by "Please, explain" narrative answer field.
‡ Presented to select groups, depending on context relevance (e.g., well-lit sidewalks is a concern in urban, but not rural areas).
§ Question 2A was presented to the Boston group, 2B to BQLI, 2C to the rest.

SC
Supplemental content to
J Med Libr Assoc. Jan;107(1):dx.doi.org/10.5195/jmla.2019.351
www.jmla.mlanet.org
© Keselman, Chase, Rewolinski, Chentsova Dutton, Kelly 2019

2. A friend tells you that several of her family members suffer from heart disease and she is worried about getting it too. Which, in your opinion, is the best response to her:
– We can't know the future, so the best thing is not to worry about it.
– You should make sure you eat well and exercise regularly.
– **You should discuss this with a doctor. (correct)**
– You should reduce the amount of stress in your life.

Knowledge of health risk factors

1. What are some health risk factors that a person CAN control?

2. What are some health risk factors that a person CANNOT control?

Knowledge of preventive health

1. Which of the following in an example of preventative health measure?
– Changing diet to include more fresh vegetables
– Getting a mammogram (a screening test for breast cancer)
– Using an inhaler to stop an asthma attack
– **A and B (correct)**
– All of the above

2. Name four diseases or health conditions that are a big concern in the United States today. For each disease or condition, suggest some preventative measures that would lower its occurrence.

Knowledge of nutrition

Look at the potato chips food label given to you by your instructor and use it to answer the following questions:

1. The majority of calories in this food come from:
– Carbohydrate
– **Fat (correct)**
– Protein
– Vitamins

2. Forty-five potato chips contain approximately:
– 160 calories
– 320 calories
– **480 calories (correct)**
– 640 calories

3. Approximately how many calories are there in a third of this bag?
– 160 calories
– 480 calories
– 1,400 calories
– **Impossible to tell from this label (correct)**

4. What percentage of the daily value of salt is contained in one portion of this food?
- 0%
- 6%
- **7% (correct)**
- Impossible to tell from this label

5–9. The five food groups are grains, proteins, vegetables, fruit, and dairy. To which food group does each of these foods belong? (Choose one: Grains, Proteins, Vegetables, Fruit, Dairy, Not Sure)
- Baked salmon **(correct: proteins)**
- Yogurt **(correct: dairy)**
- Rice **(correct: grains)**
- Apples **(correct: fruit)**
- Green beans **(correct: vegetables)**

Information evaluation skills

1. Please, explore this site: www.lasikathome.com. Imagine that a family member who is interested in vision correction found this site. She is trying to decide whether the site is good and reliable. What information would you advise her to consider in making that decision?

2. Please, take a look at this site: nei.nih.gov/health/cataract/cataract_facts. Imagine that a family member concerned about cataracts (a vision problem) found this site. She is trying to decide whether the site is good and reliable. What information would you advise her to consider in making that decision?

3. When you are looking for health information sites, what things tell you whether a site has good information or not?

Awareness of quality health information resources

1. Can you think of quality websites for someone looking for health information? Please, list the sites.

Knowledge and interest in health careers

1. Please, name as many health-related jobs as you can. For each job, select what fits best (Disagree/Not sure/Agree):
- I know what work it involves
- I know what education it requires
- I know how much it pays
- I could see myself doing that job
