# Supplementary material for: Lessons learned from multisite implementation and evaluation of Project SHARE, a teen health information literacy, empowerment, and leadership program
Source: J Med Libr Assoc. 2019 Jan 1;107(1):72–9. doi: 10.5195/jmla.2019.351 (PMC6300228; doi:10.5195/jmla.2019.351)
Supplement: Appendix C [file jmla-107-72-s003.pdf]

JMLA Journal of the Medical Library Association

SC

Supplemental content to
J Med Libr Assoc. Jan;107(1):dx.doi.org/10.5195/jmla.2019.351
www.jmla.mlanet.org
© Keselman, Chase, Rewolinski, Chentsova Dutton, Kelly 2019

Lessons learned from multisite implementation and evaluation of Project SHARE, a teen health information literacy, empowerment, and leadership program

Alla Keselman, PhD, MA; Rachel Anne Chase, MPH; Jennifer Rewolinski, BS; Yulia Chentsova Dutton, PhD; Janice E. Kelly, MLS, FMLA

APPENDIX C

Survey sections and outcome variables

Outcome variable category	Corresponding Project SHARE lesson	Sites	Survey questions and format	Outcome variables (sites with significant improvements)	Stats analysis cluster
Knowledge: community level	1.1 Intro to health disparities	All	Students review a list of social/environmental factors connected to health disparities (e.g., living in a neighborhood that has well-lit sidewalks, being part of a family that owns a car); select those that "make a person more likely to be healthy" (multiple choice); explain (open-ended narrative) those checked as contributors.	• Number of factors recognized as social determinants of health<br>• Average proportion of possible explanations per recognized health determinant*	Knowledge of health disparities and social determinants of health
Knowledge: community level	2.2 Social determinants of health	All	Students explain a health disparity affecting their community (e.g., residents of certain Boston neighborhoods are less likely to have good dental health).	• Proportion of possible reasons explaining a local disparity*	Knowledge of health disparities and social determinants of health
Knowledge: personal	3.1 Family health history	E CT, MT	Students answer multiple choice questions about the importance of knowing family health history.	• Understanding of health relevance of one's family history	Understanding of the importance of knowing one's family history
Knowledge: community level, personal	3.2 Preventive health	E CT, MT	Students list health risk factors that individuals can/cannot control.	• Knowledge of health risk factors one can control<br>• Knowledge of health risk factors one cannot control	Knowledge of health risk factors

Supplemental content to
J Med Libr Assoc. Jan;107(1):dx.doi.org/10.5195/jmla.2019.351
www.jmla.mlanet.org
© Keselman, Chase, Rewolinski, Chentsova Dutton, Kelly 2019

Outcome variable category	Corresponding Project SHARE lesson	Sites	Survey questions and format	Outcome variables (sites with significant improvements)	Stats analysis cluster
Knowledge: community level, personal	3.2 Preventive health	E CT, MT	Students list diseases that are public health concerns in the United States and preventive measures against those diseases. They also answer a multiple choice question selecting a preventive health measures from a list of options (e.g., a mammogram).	• Awareness of diseases that are public health concern in the United States (count)<br>• Average number of known preventive health measures per disease<br>• Preventive measures recognition	Knowledge of preventive health
Knowledge: personal	4.1 The importance of food labels<br>4.2 Planning of a nutritious meal	E CT, MT, CO	Students answer multiple-choice questions about a food label (e.g., determine amount of daily value of different nutrients for specified portion size); match foods to food groups.	• Knowledge of nutritional groups and the basics of food labels	Knowledge of nutrition
Skills: health information literacy	2.2 Locating and evaluation of health information	All	Students review two sites about vision, an authoritative National Eye Institute site† and a hoax site about corrective lasik eye surgery home kits‡. For each site, they respond to the following prompt: "[A family member] is trying to decide whether the site is good and reliable. What information would you advise her to consider in making that decision?" Students also list general information evaluation criteria.	• Recognition of information quality markers of a hoax site<br>• Recognition of information quality markers of an authoritative site<br>• Knowledge of general online information quality criteria	Information evaluation skills
Skills: health information literacy	2.2 Locating and evaluation of health information	All	Students name quality health information sites.	• Number of quality site mentions<br>• Number of MedlinePLUS mentions	Awareness of quality health information resources

Supplemental content to
J Med Libr Assoc. Jan;107(1):dx.doi.org/10.5195/jmla.2019.351
www.jmla.mlanet.org
© Keselman, Chase, Rewolinski, Chentsova Dutton, Kelly 2019

Outcome variable category	Corresponding Project SHARE lesson	Sites	Survey questions and format	Outcome variables (sites with significant improvements)	Stats analysis cluster
Knowledge: careers	Health careers	BQLI, CO, E CT	Students list up to ten health occupations, rate their knowledge of those careers (what each occupation involves, education it requires, average pay), respond about seeing oneself in that occupation in the future.	• Number of health occupations known<br>• Average knowledge score per known health occupation<br>• Number of health occupations of interest	Knowledge and interest in health careers

* Assessed against expert model.

BQLI=Brooklyn-Queens-Long Island; NE CO=Northeastern Colorado; SW CO=Southwestern Colorado; E CT=Eastern Connecticut; MT=Montana.

† nei.nih.gov/health/cataract/cataract_facts.

‡ www.lasikathome.com/.
