# Supplementary material for: Lessons learned from multisite implementation and evaluation of Project SHARE, a teen health information literacy, empowerment, and leadership program
Source: J Med Libr Assoc. 2019 Jan 1;107(1):72–9. doi: 10.5195/jmla.2019.351 (PMC6300228; doi:10.5195/jmla.2019.351)
Supplement: Appendix D [file jmla-107-72-s004.pdf]

JMLA Journal of the Medical Library Association

SC

Supplemental content to
J Med Libr Assoc. Jan;107(1):dx.doi.org/10.5195/jmla.2019.351
www.jmla.mlanet.org
© Keselman, Chase, Rewolinski, Chentsova Dutton, Kelly 2019

Lessons learned from multisite implementation and evaluation of Project SHARE, a teen health information literacy, empowerment, and leadership program

Alla Keselman, PhD, MA; Rachel Anne Chase, MPH; Jennifer Rewolinski, BS; Yulia Chentsova Dutton, PhD; Janice E. Kelly, MLS, FMLA

APPENDIX D

Details of statistical analysis and results

To reduce the number of comparisons while assessing significance of variables across sites, continuous outcome variables from each cluster were included in repeated measure multivariate analysis of variance (MANOVA) accounting for participants being nested within sites. If MANOVA showed significant effect of time (pretest to posttest) or of time by site interaction for a cluster, the analysis identified variables that drove the difference. In addition, we also conducted paired sample *t*-tests for within-site comparisons. As the results specify which variables reached overall significant time or interaction effects, Bonferroni correction for multiple comparisons was not applied. Categorical variables were analyzed via McNemar tests. Due to data attrition, the analyses are underpowered and, likely, overly conservative.

A. Cluster 1, Knowledge: Knowledge of health disparities and social determinants of health

In this three-variable cluster—Number of factors recognized as social determinants of health, Average proportion of possible explanations per recognized health determinant, and Proportion of possible reasons explaining a local disparity—repeated measure MANOVA indicated significant effect of site, Wilks' Λ=0.40, F(18,124.94)*=2.63, p=0.01. Follow-up univariate tests showed that while Average proportion of possible explanations per recognized health determinant differed across sites, F(6,46)=8.44, p<0.01, there were no differences for the other outcome variables.

There was no significant overall pretest-posttest increase across measures, Wilks' Λ=0.95, F(3,44)=0.85, *ns.* However, there was a significant interaction between site and time of measurement, Wilks' Λ=0.38, F(18, 124.94)=2.82, p<0.01, with univariate tests indicating that the magnitude changes in Average proportion of possible explanations per recognized health determinant differed by site and contributed to the overall effect, F(6,47)=5.75, p<0.01, while there were no significant site by pretest-posttest comparison interactions for the other 2 variables. Paired sample *t*-test comparison of pretest-posttest group means was performed post-hoc to examine the results site-by-site (Table 1).

* Degrees of freedom for a repeated-measure nested MANOVA often involve fractions.

JMLA Journal of the Medical Library Association

Supplemental content to
J Med Libr Assoc. Jan;107(1):dx.doi.org/10.5195/jmla.2019.351
www.jmla.mlanet.org
© Keselman, Chase, Rewolinski, Chentsova Dutton, Kelly 2019

Table 1 Knowledge of health disparities and social determinants of health cluster outcomes

Site	Number of factors recognized as social determinants of health*, M-pre(STD-pre)–M-post (STD-post), *p*	Average proportion of possible explanations per recognized health determinant, M-pre(STD-pre)–M-post (STD-post), *p*†	Proportion of possible reasons explaining a local disparity, M-pre(STD-pre)–M-post (STD-post), *p*
Boston	NSI‡	NSI	NSI
BQLI	4.63(2.26)–6.75(1.04), *p*<0.018	NSI	NSI
MT	NSI	NSI	NSI
NE CO	NSI	NSI	NSI
SW CO	NSI	0.04(05)–0.30(0.09), *p*<0.001	0.02(0.05)–0.15 (.05), *p*<0.012
E CT	4.35(2.01)–6.30(2.13), *p*<0.008	NSI	0.023(0.10)–0.029(.13), *p*<0.028

* Maximum possible score 8.
† Significant overall time × site interaction for this variable.
‡ No statistically significant improvement.
BQLI=Brooklyn-Queens-Long Island; NE CO=Northeastern Colorado; SW CO=Southwestern Colorado; E CT=Eastern Connecticut; MT=Montana.

B. Cluster 2, Knowledge: Awareness of the importance of knowing one's family health history

Awareness of health relevance of one's family history section (taken by Montana [MT] and Eastern Connecticut [E CT] participants only) consisted of 2 questions that were summed into 1 variable. There was no significant overall pretest-posttest increase, F(1,25)=1.06, *ns*. However, there was a significant interaction between site and time of measurement, F(2, 25)=3.37, *p*=0.05. Paired samples *t*-test showed a significant pretest-posttest difference in the E CT, from M=1.30(STD=0.73) to M=1.85(STD=.037), *p*<0.008, but not the MT group.

C. Cluster 3, Knowledge: Knowledge of health risk factors

This 2-variable cluster (MT and E CT participants only) consisted of 2 variables, Knowledge of health risk factors one can control and Knowledge of health risk factors one cannot control. Repeated measure MANOVA indicated significant differences across sites, Wilks' Λ=0.63, F(4,48)=3.13, *p*=0.02. Follow-up univariate tests showed that Knowledge of health risk factors one can control differed across sites, F(2,25)=4.58, *p*=0.02, while Knowledge of health risk factors one cannot control did not. There was no significant pretest-posttest increase across measures, Wilks' Λ=0.99, F(2, 24)=1.44, *ns,* and no significant interaction between site and time of measurement, Wilks' Λ=0.77, F(4, 48)=1.69, *ns*.

T-test analyses suggested that, in both cases, the change was significant for the E CT, but not the MT group. With regard to the factors one can control, E CT group's mean knowledge score increased from 0.75(STD=0.79) on the pretest to 1.25(STD=0.91) on the posttest (*p*<0.008). The corresponding knowledge scores for factors one cannot control changed from 0.30(STD=0.57) on the pretest to 0.85(STD=0.67) on the posttest (*p*<0.001).

JMLA Journal of the Medical Library Association

SC

Supplemental content to
J Med Libr Assoc. Jan;107(1):dx.doi.org/10.5195/jmla.2019.351
www.jmla.mlanet.org
© Keselman, Chase, Rewolinski, Chentsova Dutton, Kelly 2019

D. Cluster 4, Knowledge: Knowledge of preventive health

A repeated measure MANOVA (MT and E CT participants only) with 2 outcome variables, Awareness of diseases that are public health concerns in the United States (Count) and Average number of known preventive health measures per disease, indicated no overall significant differences across sites, Wilks' $\Lambda=0.60$, $F(4,34)=2.50$, *ns.* There was significant pretest-posttest increase across measures, Wilks' $\Lambda=0.48$, $F(2,17)=9.35$, $p<0.01$. Univariate tests indicated that pretest-posttest increase was significant for the Awareness of disease (Count), $F(1, 18)=6.88$, $p=0.02$, but not Average number of known preventive health measures per disease.

There was also a significant interaction between site and time of measurement, Wilks' $\Lambda=0.36$, $F(4, 34)=5.72$, $p<0.01$. Univariate tests indicated that the magnitude changes in both the Awareness of disease (Count), ($F(2,18)=5.00$, $p=0.02$), and Average number of known preventive health measures per disease, ($F(2,18)=3.46$, $p=0.05$), differed by site and contributed to the overall effect. *T*-test analysis suggested that the effect of the Awareness of disease (Count) was significant for the MT group (pretest-posttest mean increase from 2.75(STD=1.04) to 3.75(STD=0.46), $p<0.033$), but not the E CT group.

As a single multiple-choice question variable, Preventive measures recognition, could not be included in the MANOVA analysis and was analyzed separately via McNemar test. The proportion of students correctly answering this question differed significantly between pretest and posttest across groups. Examination of this effect site-by-site showed that the contrast was significant for the E CT group, in which the number of correct responses changes from 10% on the pretest to 65% on the posttest ($p<0.0001$).

E. Cluster 5, Knowledge: Knowledge of nutrition

Nine Knowledge of nutrition multiple-choice questions were summed to constitute this outcome variable (MT and E CT participants only). There was no overall significant pretest-posttest increase, $F(1,25)=0.04$, *ns.* However, there was a significant interaction between site and time of measurement, $F(2, 25)=7.59$, $p<003$. Paired *t*-tests revealed that the effects were significant for the E CT group, where the mean score increased from 7.05(STD=1.19) on the pretest to 8.80(STD=0.41) on the posttest ($p<0.0001$).

F. Cluster 6, Health information literacy: Information evaluation skills

A repeated measure MANOVA with the 3 outcome variables in this cluster—Recognition of information quality markers of a hoax site, Recognition of information quality markers of an authoritative site, and Knowledge of general online information quality criteria—indicated overall significant differences across sites, Wilks' $\Lambda=0.29$, $F(18,124.94)=3.76$, $p<0.01$. Follow-up univariate tests showed that difference held true for all 3 variables: Recognition of information quality markers of a hoax site, $F(6,46)=3.92$, $p<0.01$; Recognition of information quality markers of an authoritative site, $F(6,46)=2.55$, $p=0.03$; and Knowledge of general online information quality criteria, $F(6,46)=5.97$, $p<0.01$.

There was significant pretest-posttest increase across measures, Wilks' $\Lambda=0.76$, $F(3,44)=4.62$, $p<0.01$. Univariate tests indicated that pretest-posttest increase was significant for the Recognition of information quality markers of an authoritative site, $F(1, 46)=9.50$, $p<0.01$, and Knowledge of general online information quality criteria $F(1,46)=7.61$, $p<0.01$, but not Recognition of information quality markers of a hoax site score. There was also a significant interaction between site and time of measurement, Wilks' $\Lambda=0.51$, $F(18,124.94)=1.86$, $p=0.02$. Univariate tests indicated that the magnitude changes in Recognition of information quality markers of an authoritative site, $F(6, 46)=3.44$, $p<0.01$, but not the other two variables, differed by site and contributed to the overall effect. Results of paired sample *t*-test comparison of pretest-posttest group means are reported in Table 2.

SC
Supplemental content to
J Med Libr Assoc. Jan;107(1):dx.doi.org/10.5195/jmla.2019.351
www.jmla.mlanet.org
© Keselman, Chase, Rewolinski, Chentsova Dutton, Kelly 2019

Table 2 Information evaluation skills cluster outcomes

Site	Hoax site evaluation score*, M-pre(STD-pre)–M-post (STD-post), *p*	Authoritative site evaluation score†‡§, M-pre(STD-pre)–M-post (STD-post), *p*	General website evaluation criteria score†‡, M-pre(STD-pre)–M-post (STD-post), *p*
Boston	0.38(0.74)–1.63(0.92), *p*<0.005	0.75(0.89)–2.38(0.92), *p*<.003	0.63(0.92)–2.50(0.54), *p*<0.001
BQLI	NSI**	NSI	1.00(0.53)–1.88(0.99), *p*<0.041
MT	NSI	NSI	0.75(0.71)–1.63(0.92), *p*<0.021
NE CO	NSI	NSI	NSI
SW CO	NSI	0.00(0.00)–1.17(0.98), *p*<0.017	0.00(0.00)–1.67(1.21), *p*<0.02
E CT	NSI	NSI	0.40(0.60)–0.95(1.00), *p*<0.045

* Maximum possible score 6.
† Maximum possible score 5.
‡ Significant overall time effect for this variable.
§ Significant overall time-site interaction for this variable.
** No statistically significant improvement.

G. Cluster 7, Health information literacy: Awareness of health information resources

Because the 2 variables in this group were of different types (continuous versus binary), they were analyzed separately. For the Awareness of quality health information sites, there was no overall significant pretest-posttest increase, $F(1,46)=0.75$, *ns,* and no significant interaction between site and time of measurement, Wilks' $\Lambda=0.80$, $F(6,46)=1.88$, *ns*. Paired samples *t*-test was significant only for the E CT group, where the number of quality health-related sites increased from 0.30(STD=0.66) on the pretest to 2.20(STD=1.80) on the posttest, $p<0.0001$. In addition, McNemar test suggested significant overall improvement in the proportion of Number of MedlinePlus mentions by participants, with significant improvements in Brooklyn-Queens-Long Island (BQLI) (no one on the pretest, 88% of the participants on the posttest, $p<0.004$) and E CT sites (no one on the pretest, 35% of the participants on the posttest, $p<0.004$), but not at the other sites.

H. Cluster 8, Knowledge: Knowledge of and interest in health careers

A repeated measure MANOVA with the 3 outcome variables in this cluster (BQLI, Northeastern Colorado [NE CO], Southwestern Colorado [SW CO], and E CT)—Number of health occupations known, Average knowledge score per known health occupation, and Number of health occupations of interest—indicated no overall significant differences across sites. The overall pretest-posttest increase across measures was not significant, Wilks' $\Lambda=0.92$, $F(3,27)=0.81$, *ns,* but there was a significant interaction between site and time of measurement, Wilks' $\Lambda=0.40$, $F(12,71.73)=2.50$, $p<0.01$. Univariate tests indicated that the magnitude changes in the Number of Health Occupations of Interest, $F(4,29)=3.66$, $p=0.02$, differed by site, but not the other 2 variables, and contributed to the overall effect. Results of paired sample *t*-test comparison of pretest-posttest group means are reported in Table 3.

JMLA Journal of the Medical Library Association

Supplemental content to
J Med Libr Assoc. Jan;107(1):dx.doi.org/10.5195/jmla.2019.351
www.jmla.mlanet.org
© Keselman, Chase, Rewolinski, Chentsova Dutton, Kelly 2019

Table 3 Knowledge and interest: health careers

Site	Number of health occupations known, M-pre(STD-pre)–M-post (STD-post), *p*	Average knowledge score per known health occupation, M-pre(STD-pre)–M-post (STD-post), *p*	Number of health occupations of interest, M-pre(STD-pre)–M-post (STD-post), *p*
BQLI	NSI	NSI	1.13(1.36)–2.62(1.92), $p<0.048$
NE CO	NSI	NSI	NSI
SW CO	2.00(1.73)–5.00(1.00), $p<0.011$	NSI	NSI
E CT	2.74(1.70)–4.21(1.58), $p<0.002$	1.67(0.77)–2.34(0.49), $p<0.001$	NSI
